# Drug dosing in the critically ill obese patient: a focus on medications for hemodynamic support and prophylaxis

**DOI:** 10.1186/s13054-021-03495-8

**Published:** 2021-02-23

**Authors:** Brian L. Erstad, Jeffrey F. Barletta

**Affiliations:** 1 Department of Pharmacy Practice and Science, College of Pharmacy, University of Arizona, 1295 N Martin Ave, PO Box 210202, Tucson, AZ 85721 USA; 2 Department of Pharmacy Practice, College of Pharmacy, Midwestern University, 19555 N 59th Ave, Glendale, AZ 85308 USA

**Keywords:** Critical illness, Obesity, Pharmacokinetics, Drug dosing

## Abstract

Medications used for supportive care or prophylaxis constitute a significant portion of drug utilization in the intensive care unit. Evidence-based guidelines are available for many aspects of supportive care but drug doses listed are typically for patients with normal body habitus and not morbid obesity. Failure to account for the pharmacokinetic changes that occur with obesity can lead to an incorrect dose and treatment failure or toxicity. This paper is intended to help clinicians design initial dosing regimens in critically ill obese patients for medications commonly used for hemodynamic support or prophylaxis. A detailed literature search of medications used for supportive care or prophylaxis listed in practice guidelines was conducted with an emphasis on obesity, pharmacokinetics and dosing. Relevant manuscripts were reviewed and strategies for dosing are provided. For medications used for hemodynamic support, a similar strategy can be used as in non-obese patients. Similarly, medications for stress ulcer prophylaxis do not need to be adjusted. Anticoagulants for venous thromboembolism prophylaxis, on the other hand, require an individualized approach where higher doses are necessary.

## Introduction

Medications for supportive care and prophylaxis constitute a large proportion of drug use in the intensive care unit (ICU). In fact, evidence-based guidelines exist for many of the therapies considered “routine care” and form the basis for checklists and standardization of therapy. The drug doses that are included, however, are often formulated for patients with normal body habitus and do not account for the pharmacokinetic variability encountered with obesity. This is concerning because clinical trials that provide efficacy and safety data for the ICU rarely include obese individuals thereby presenting a unique challenge for bedside clinicians when designing a dosing regimen. Most data in this area are from studies that utilize pharmacokinetic variables, surrogate markers for efficacy or physicochemical characteristics. Nevertheless, clinicians must still make important dosing decisions at the bedside despite the limited amount of data that are available.

**Fig. 1:**
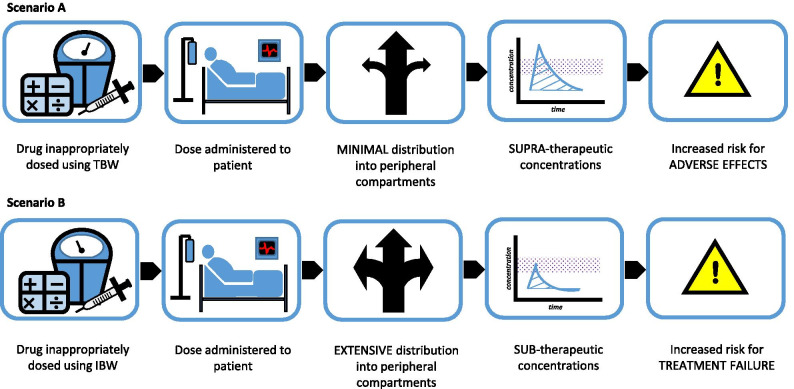
Consequences of using an incorrect weight metric when dosing weight-based medications in obese patients

Recent data from the CDC indicate the prevalence of obesity (BMI ≥ 30 kg/m^2^) among US adults is 42.4% [1]. Furthermore, there is an alarming increase in the prevalence of severe obesity (BMI ≥ 40 kg/m^2^) which has risen from 4.7% in 1999 to 9.2% in 2018. Unfortunately, this trend has not been recognized with regard to the availability of obesity-specific dosing information in product labeling. One study, published in 2020, stated only 30% of medications evaluated had some reference to a weight descriptor in the dosing information compared to 27% reported in a similar study conducted about 10 years prior [2, 3]. This is problematic because the use of an incorrect weight metric, for weight-based dosing, could lead to treatment failure (in the event of subtherapeutic levels) or drug toxicity (caused by supratherapeutic levels) (Fig. 1). Knowledge of the most appropriate weight-metric for each medication is essential to optimize outcomes with drug therapy in the critically ill obese patient.

The weight metric used to characterize weight status is body mass index (BMI). Body mass index, however, is not commonly used for drug dosing. The weight metrics most commonly referenced when dosing medications in the ICU are total body weight or some alternative, such as ideal body weight (IBW), lean body weight (LBW) or adjusted body weight (Table 1). Ideal body weight characterizes weight based on height and gender. It was formulated more than 60 years ago using actuarial data based on the premise that for a given height, there was an ideal weight [4]. Ideal body weight does not account for differences in body composition or the increases in absolute lean mass that typically accompany obesity. Thus, it is inherently flawed as a surrogate for fat-free mass. Lean body weight appears to be the best representation of fat-free mass [5]. These equations, however, are prone to calculation errors so software programs are recommended. Adjusted body weight using a correction factor (i.e., a fraction of the difference between total and ideal body weight) is commonly used for drug dosing, and these equations are well known by most practitioners. Adjusted body weight is roughly equivalent to LBW and for the purposes of this paper will be considered a surrogate (for LBW) because of familiarity and ease of calculation.

**Table 1:** Common weight measures used to estimate size when dosing medications in obese patients

Weight measure	Equation
Body mass index (kg/m^2^)	TBW/height (m)^2^
Ideal body weight (kg) [74]	Males: 50 kg + 2.3 kg/inch for height over 5 feet Females: 45.5 kg + 2.3 kg/inch for height over 5 feet
Lean body weight (kg) [5]	Males: (9270 × TBW) / (6680 + 216 × BMI) Females: (9270 × TBW) / (8780 + 244 × BMI)
Adjusted body weight (kg) [75]	CF (TBW − IBW) + IBW, where CF = correction factor (usually 0.4)

Designing dosing regimens in the critically ill obese patient requires a detailed understanding of the physicochemistry of the medication, and the impact obesity has (coupled with critical illness) on physiology and drug pharmacokinetics [6]. Because of the tremendous variability observed, an individualized dosing approach is preferred [7]. Generalized clinical pearls exist to assist with dosing in this challenging population (Table 2) but specific dosing recommendations to guide clinicians are limited. The purpose of this paper is to assist clinicians with dosing regimens for medications commonly used as part of the supportive care and prophylaxis in critically ill obese patients.

**Table 2:** Generalized clinical pearls for crafting medication doses in the setting of extreme obesity

• Seek consistency within and between all healthcare professionals involved in size estimates	
• Clearly document instrument (e.g., type of scale) and methods used (e.g., clothes on or off) for weight measurement	
• Document when estimates or patient-stated weights are utilized	
• When using total body weight, do not change the weight used for calculations (due to weight gain or loss) after therapy has been initiated	
• Clinical monitoring of the individual patient’s response to therapy should supersede data from pharmacokinetic studies	
• Pharmacokinetic parameters (volume of distribution, clearance) are typically greater and with more variability in critically ill versus non-critically ill patients	
• Assess for dose-proportionality using pharmacokinetic studies that report volume of distribution and clearance in obese and non-obese individuals	
• The duration of action of a single dose (i.e., a loading dose) is generally a function of volume of distribution rather than clearance	
• Always assess the benefits and risks of the dosing regimen especially when using a larger dose or total body weight for calculations (i.e., is there a narrow therapeutic index or dose-related adverse effects)	
• Use technology or automated dosing calculators to minimize calculation errors	
• Even with medications that can be rapidly titrated to effect, using an inappropriate weight metric for weight-based dosing can lead to adverse effects upon initiation or maximum doses above-recommended values	

## Methodology

The medications reviewed for evaluation consisted of those used for supportive care or prophylaxis mentioned in evidence-based guidelines. Published guidelines from the following therapeutic domains were screened: pain, agitation, delirium, neuromuscular blockade, hemodynamic support in sepsis, stress ulcer prophylaxis and venous thromboembolism prophylaxis [8–12]. Antimicrobials were not included given the overarching theme of this manuscript coupled with the availability of other manuscripts providing dosing recommendations in this area [13–15]. Furthermore, thorough reviews providing recommendations for medications used for pain, agitation, delirium and neuromuscular blockade are also available and the reader is referred to these texts [16, 17]. Thus, the remaining areas included were medications for hemodynamic support in shock (i.e., vasopressors and corticosteroids), stress ulcer prophylaxis and venous thromboembolism.

A detailed literature search was performed using PubMed from inception to July 2020, using search terms from the following three categories: (1) obesity: "Obesity"[Mesh] OR "Overweight"[Mesh] OR "body composition"[MeSH Terms] OR "extreme obesity" OR "body weight change*" OR "body size" OR "body fat" OR "body fatness" (2) pharmacokinetics and dosing: "Drug Monitoring"[Mesh] OR "Dose–Response Relationship, Drug"[Mesh] OR "pharmacokinetic" OR "pharmacokinetic considerations " OR "drug dosing" OR "drug dose" OR "therapeutic drug monitoring" OR "drug monitoring" and (3) the specific drug in question. The results from the primary literature search were reviewed and pertinent articles were retained. Bibliographies were reviewed for any articles that may have been missed by the primary literature search. Non-English articles and animal studies were not included. The focus will be on adult patients with more severe forms of obesity (i.e., BMI ≥ 40 kg/m^2^), since such patients are typically limited in numbers in the studies used to formulate product labeling information. A comprehensive, online database was consulted for drug physicochemical properties (e.g., octanol–water partition coefficient (log *P*)) [18]. This database provides detailed drug data (e.g., chemical, pharmacological and pharmaceutical) and comprehensive drug target information (e.g., sequence, structure and pathway) with more than 14,000 drug entries. Suggestions were then formed using the available data based on the following prioritization strategy: studies evaluating clinical outcomes, pharmacokinetics, adverse effect profiles and physicochemical properties. Because of the heterogeneity of study outcomes (i.e., pharmacokinetic-related, clinical outcome, etc.) and the expected lack of information for many of the medications included, advanced statistical techniques such as meta-analysis were not performed.

### Vasopressors

All of the commonly used vasopressors are hydrophilic as indicated by negative log *P* values, so distribution is typically limited at most to the extracellular fluid compartment. Small volumes of distribution combined with rapid clearance values results in short half-lives for these agents typically necessitating their administration as continuous intravenous infusions. Irrespective of obesity, there is substantial variability in the pharmacokinetics and pharmacodynamics of vasoactive agents (dopamine, dobutamine, epinephrine, norepinephrine and angiotensin II) when used in critically ill patients [19–23]. These physicochemical, pharmacokinetic and pharmacodynamic characteristics argue for the use of non-weight-based dosing regimens, or for weight-based dosing regimens with the use of an IBW or lean body mass descriptor.

In multicenter, retrospective studies evaluating the outcomes of patients with severe infections including septic shock, obese patients received significantly lower weight-based doses of fluids, norepinephrine and other vasopressors compared to normal-weight patients with either no change or lower overall mortality [24–26]. Further, data from single-center, retrospective evaluations investigating weight descriptors for dosing vasopressor medications in critically ill, obese patients demonstrate substantial inter-patient variability in response to vasopressor administration with no consistent weight-based, dose–response relationship [27–31].

Summary: For vasopressors administered as continuous infusions, either a non-weight-based dosing regimen, or a weight-based dosing regimen using an ideal or adjusted body weight is suggested for initial doses in obese patients. If a weight-based method is chosen, seek consistency between using ideal or adjusted body weight across different vasopressor agents (e.g., norepinephrine and dopamine) to minimize error risk.

### Corticosteroids

The majority of studies published to date evaluating relationships between corticosteroids and obesity concern hypothalamic–pituitary–adrenal axis regulation and cortisol activity [32]. In one of the few pharmacokinetic studies involving corticosteroids and obese subjects, methylprednisolone pharmacokinetics were compared in 6 obese and 6 non-obese males [33]. Obese patients received a dose of 0.6 mg/kg while non-obese subjects received a fixed dosage of 40 mg (approximately 0.5 mg/kg based on reported total body weight). Volume of distribution was closely related to IBW suggesting limited distribution into adipose tissue. The authors concluded doses based on IBW and not total body weight were recommended. In a second study prednisolone disposition was assessed in 8 obese and 4 normal-weight (i.e., actual weight equaled IBW) men after a single intravenous injection of 33 mg [34]. The obese compared to the normal-weight subjects had proportional increases in volume of distribution (approximately 20%) and clearance (approximately 35%) which were less than the proportional differences in actual body weight in the obese subjects (62% above IBW). The differences in the pharmacokinetic parameters in the obese subjects are more consistent with those expected from hydrophilic medications that primarily distribute into lean tissue, rather than the dose proportional increases expected with more lipophilic agents (e.g., log *P* values between 1 and 2) like corticosteroids. Additionally, corticosteroids easily pass through cell membranes to bind to cytosolic glucocorticoid receptors that are present in almost all body cells, so the relationship between pharmacokinetic and pharmacodynamics effects is complex [35]. All of these issues complicate the choice of an appropriate size descriptor when considering weight-based dosing regimens. In adult critically ill patients, current guidelines recommend intravenous doses of hydrocortisone equivalents of less than 400 mg daily for hospitalized patients with community-acquired pneumonia or septic shock unresponsive to fluids and vasopressors, and intravenous doses of methylprednisolone of 1 mg/kg daily for patients with early moderate to severe acute respiratory distress syndrome (ARDS) [9, 11]. These doses result in supraphysiological levels of corticosteroid in terms of cortisol equivalents [36].

Summary: For non-weight-based dosing of hydrocortisone in patients with community-acquired pneumonia or septic shock unresponsive to fluids and vasopressors, intravenous doses of hydrocortisone in obese patients should be the same as those used in non-obese patients. For weight-based dosing of methylprednisolone for patients with ARDS, the use of an ideal or adjusted body weight is suggested for weight-based dosing in obese patients, particularly in patients with more severe forms of obesity (e.g., BMI of 40 kg/m^2^ or greater).

### Stress ulcer prophylaxis

Acid suppressive therapy is routinely administered to critically ill patients for the prevention of clinically important gastrointestinal bleeding (CIB) due to stress ulcers. The agents most commonly chosen for stress ulcer prophylaxis (SUP) are the proton pump inhibitors (PPI) followed by histamine-2-receptor-antagonists (H2RA) [37]. Unfortunately, clinical trials comparing effectiveness in obese patients are lacking. In the most recent prospective trials evaluating SUP, weight is not reported, making the impact of obesity on outcomes difficult to determine [38–40].

The PPIs and H2RA’s are both considered acceptable therapy for the provision of SUP but there are some differences in their pharmacokinetic profiles that could be affected by obesity. Proton pump inhibitors are highly lipophilic as prodrugs, which promote distribution into adipose tissue. The H2RA’s, on the other hand, are hydrophilic compounds and poorly distribute into fat. Pantoprazole, lansoprazole, omeprazole and esomeprazole are metabolized through the cytochrome P-450 (CYP) system. (Rabeprazole is metabolized by a non-enzymatic process.) Both animal and human studies have shown a correlation between increased liver fat content and decreased CYP activity [41]. This relationship has not been quantified across varying degrees of obesity; thus, the clinical significance remains unknown. Body mass index has been associated with nonalcoholic fatty liver disease in a near-linear relationship [42]. Histamine-2-receptor antagonists are metabolized through non-CYP pathways and primarily eliminated renally [43].

There are limited studies evaluating the pharmacokinetics of acid suppressive medications in obesity and none are specific to ICU patients for the provision of SUP. Most data originate from pharmacokinetic studies conducted in healthy volunteers or symptom-related outcomes in patients with gastroesophageal reflux disease (GERD). Furthermore, few patients in these studies had more extreme forms of obesity (e.g., BMI > 40 kg/m^2^). Extrapolation of these results to the ICU population can be difficult due to the differences in pathophysiological features (between CIB due to stress ulceration and GERD) and alterations that occur in critical illness. One study evaluated the effect of obesity on intragastric pH following a single dose of PPI (pantoprazole or rabeprazole) in patients with GERD [44]. There was no correlation between BMI and the total time with pH > 4. Other studies have evaluated outcomes in GERD patients such as esophageal pH, heartburn symptoms and healing of erosive esophagitis [45–47]. Overall, these studies have demonstrated no differences based on BMI. Weight-based dosing of PPI’s has been evaluated in the pediatric population. These studies have revealed PPI exposure correlates best with lean body weight dosing as opposed to actual body weight [48, 49]. Collectively, these data reveal that despite some of the theoretical pharmacokinetic concerns with PPI’s in obesity, obesity has minimal impact on PPI-related pharmacodynamics.

Similar to PPI’s, the pharmacokinetic parameters of H2RA’s are not largely affected by obesity [50–52]. In one study of surgical patients with BMI ≥ 35 kg/m^2^, preoperative ranitidine was effective in increasing gastric pH (6.1 ± 1.2) compared to unmedicated controls (3.5 ± 1.6) [53]. Standard doses of H2RA’s therefore seem to be adequate.

Summary: Standard, non-weight-based doses for both H2RA’s and PPI’s are appropriate for stress ulcer prophylaxis in obese critically ill patients.

### Venous thromboembolism prophylaxis

Obesity is a well-known risk factor for venous thromboembolism (VTE) in both critically ill and non-critically ill patients. In a risk factor analysis using data from a large randomized thromboprophylaxis trial, each 10-point increase in BMI was associated with a significant increase in both proximal deep vein thrombosis [HR (95% CI) 1.25 (1.06–1.46)] and pulmonary embolism [HR (95% CI) 1.37 (1.02–1.83)] [54]. Prophylaxis in the ICU is typically provided with low molecular weight heparin or unfractionated heparin using a fixed dosing strategy as recommended by the package insert. Standard dosing strategies, however, may be inadequate as several studies have demonstrated an inverse linear relationship between total body weight and anti-Xa activity [55, 56]. Unfortunately, the majority of the data are in the bariatric surgery population, so do not necessarily account for the pharmacokinetic variability observed in the critically ill that results from a variety of factors including the use of vasopressors and tissue edema [57, 58]. Furthermore, there are few studies focused on clinical outcomes such as VTE incidence; instead, most endpoints were directed toward surrogate markers (e.g., anti-Xa levels) [59–69]. One retrospective, before-after study compared VTE rates using two enoxaparin dosing regimens (30 mg or 40 mg subcutaneous twice daily) in a cohort of bariatric surgical patients [67]. Postoperative VTE was significantly lower with the higher dosing regimen (5.4% vs. 0.6%, *p* < 0.01). There was no difference in the incidence of hemorrhage. A second retrospective study evaluated the efficacy and safety of enoxaparin and heparin in patients who weighed at least 100 kg [69]. In this study, patients were stratified according to receipt of a standard or high dose of anticoagulant (80 mg/day of enoxaparin or 22,500 units/day of unfractionated heparin). In the cohort of patients with a BMI of at least 40 kg/m^2^, the VTE rate was 1.48% with standard dosing compared to 0.77% in the high-dose group [OR (95% CI) 0.52 (0.27–1.00);*p* = 0.05]. Similar to the previous study, no difference in hemorrhage was noted.

In light of the evidence surrounding low molecular weight heparin dosing in obesity, several alternative approaches have been evaluated based on anti-Xa levels. There is wide disparity in the doses of low molecular weight heparin utilized, the patient populations studied and the degree of obesity present among the patients. In one study specific to critically ill patients, 23 surgical ICU patients with a mean BMI of 46.4 ± 11.7 kg/m^2^ and weight of 137 ± 37 kg reported anti-Xa levels following a 0.5 mg/kg twice daily enoxaparin regimen [62]. Initial anti-Xa levels were in the appropriate range (0.2–0.5 IU/ml) in 91% of patients and none experienced major bleeding. A second retrospective study evaluated weight-based dosing of enoxaparin (0.5 mg/kg twice daily) in obese trauma patients [59]. In this study, the median BMI and weight was 35.3 kg/m^2^ and 113 kg, respectively. Target anti-Xa levels (0.2–0.6 IU/ml) were achieved in 86% of patients and no patients experienced a bleeding event. A third trial randomized hospitalized medical patients to receive enoxaparin 40 mg daily, 0.4 mg/kg daily or 0.5 mg/kg daily [61]. The average BMI in each of the study cohorts exceeded 60 kg/m^2^ while weight was greater than 170 kg (range 115–256 kg). The primary outcome was achievement of target anti-Xa level (0.2–0.5 IU/ml), which was reached significantly more often in the 0.5 mg/kg/day group compared to the other regimens. The incidence of subtherapeutic anti-Xa levels was 87%, 36% and 18% for the fixed (non-weight-based), 0.4 mg/kg and 0.5 mg/kg regimens, respectively. Finally, one prospective trial evaluated a BMI-stratified dosing approach in a cohort of bariatric surgery patients [60]. Patients with a BMI ≤ 50 kg/m^2^ received an enoxaparin dosage of 40 mg twice daily while patients in excess of 50 kg/m^2^ received 60 mg twice daily. The average BMI in each of the groups was 44.9 ± 3.7 kg/m^2^ and 57.4 ± 6.4 kg/m^2^ while weight was 126 ± 19 kg and 161 ± 27 kg, respectively. Subtherapeutic anti-Xa levels were observed in 21% of the patients who received a 40 mg dose and 14% of patients who received a 60 mg dose. There were no patients in the 40 mg cohort who were supratherapeutic but 17% were supratherapeutic in the 60 mg group. Bleeding was not associated with a high Xa-level. For a descriptive evaluation of these and other studies, the reader is referred to Additional file 1.

Summary: Critically ill obese patients who receive low molecular weight heparin require a higher dosage for VTE prophylaxis than patients who are not obese. Most data are with enoxaparin and the only dosing regimen associated with a reduction in VTE rate is 40 mg twice daily. In patients with more extreme forms of obesity (i.e., BMI ≥ 50 kg/m^2^), higher doses may be necessary. There is wide disparity in the dosing regimens suggested for these patients including a BMI-stratified approach (60 mg twice daily, equivalent to approximately 0.4 mg/kg/dose based on reported weights) to a weight-based approach ranging from 0.5 mg/kg once daily to 0.5 mg/kg twice daily. For patients with a BMI exceeding 40 kg/m^2^, enoxaparin 40 mg twice daily is appropriate. For patients with a BMI ≥ 50 kg/m^2^, a weight-based approach of 0.4–0.5 mg/kg twice daily based on total body weight is suggested. Given the lack of consistency with dosing suggestions across pharmacokinetic studies, anti-Xa monitoring seems reasonable in this population.

Unfractionated heparin dosing has also been evaluated in obese hospitalized patients (Additional file 1) [70–72]. Patanwala, et al. compared VTE rates in obese (BMI ≥ 30 kg/m^2^) and non-obese (BMI < 30 kg/m^2^) populations who received heparin 5000 units three times daily [71]. There were 5110 patients assessed (approximately 26% were critically ill) and no difference in VTE rate was noted (obese, 0.7% vs. non-obese, 0.6%; *p* = 0.7). Joy, et al. compared heparin doses of 7500 units with 5000 units every 8 h in patients who weighed more than 100 kg [70]. Approximately 37% were admitted to an ICU. Overall, there was no difference in VTE rate between the high-dose and low-dose groups (3% vs. 1.5%, *p* = 0.14). Furthermore, in the patients with a BMI ≥ 40 kg/m^2^, VTE rates were 3% (high-dose) and 2% (low-dose), *p* = 0.43. Next, unfractionated heparin dosing was evaluated in a retrospective study of neurocritical care patients [72]. Patients who weighed over 100 kg were stratified based on receipt of a traditional (5000 units every 8 h) or high (7500 units every 8 h) heparin dose. There were 398 patients included with an average weight of 116 kg (traditional dose) and 123 kg (high dose). The incidence of VTE was 9.3% and 5.7% (*p* = 0.2) for the traditional and high heparin doses, respectively. There was no difference in major bleeding events (11% vs. 14%, *p* = 0.33). Finally, high-dose unfractionated heparin (7500 units every 8 h) was compared to enoxaparin 40 mg every 12 h in a retrospective study of obese hospitalized patients (mean BMI = 49.5 ± 8.9 kg/m^2^) [73]. No difference in VTE rate was reported but major bleeding events were higher with unfractionated heparin [OR(95% CI) 1.85 (1.07–3.13)].

Summary: Critically ill obese patients who receive unfractionated heparin for VTE prophylaxis appear to have equal benefit with traditional and high-dosing regimens. When unfractionated heparin is utilized in this population, 5000 units every 8 h is appropriate. In patients with more extreme forms of obesity (BMI ≥ 50 kg/m^2^), 7500 units every 8 h can be considered.

## Conclusion

High-level evidence describing dosing of supportive care medications in obesity is lacking. Many of the medications used for supportive care can be dosed using a similar strategy to that observed in non-obese patients (e.g., vasopressors, corticosteroids and acid-suppressants). Anticoagulants for VTE prophylaxis, on the other hand, require an individualized approach. Therapeutic drug monitoring should be used where available. Further research is necessary to guide medication dosing in obese, critically ill patients.

## Supplementary Information

**Additional file 1:** Pharmacokinetic and clinical trials involving low molecular weight heparin and unfractionated heparin.

## Data Availability

Not applicable.

## Abbreviations

BMI: Body mass index
CIB: Clinically important bleeding
GERD: Gastroesophageal reflux disease
H2RA: Histamine 2 receptor antagonist
IBW: Ideal body weight
ICU: Intensive care unit
LBW: Lean body weight
PPI: Proton pump inhibitor
SUP: Stress ulcer prophylaxis
VTE: Venous thromboembolism
