# Supplementary material for: Drug dosing in the critically ill obese patient: a focus on medications for hemodynamic support and prophylaxis
Source: Crit Care. 2021 Feb 23;25:77. doi: 10.1186/s13054-021-03495-8 (PMC7901103; doi:10.1186/s13054-021-03495-8)
Supplement: Supplementary file 1 — Additional file 1: Pharmacokinetic and clinical trials involving low molecular weight heparin and unfractionated heparin. [file 13054_2021_3495_MOESM1_ESM.docx]

**Drug Dosing in the Critically Ill Obese Patient- A focus on medications for hemodynamic support and prophylaxis**

Additional files

**Additional File 1.**

Pharmacokinetic and clinical trials involving low molecular weight heparin and unfractionated heparin

| Reference | Treatment | Control | Results | Comments |
| --- | --- | --- | --- | --- |
| *Low-molecular weight heparin: Fixed-dosing strategies* | | | | |
| Scholten, 2002 (67) | N=389 patients with average BMI=50.4 kg/m^2^ undergoing bariatric surgery; enoxaparin 40 mg BID | N=92 patients with average BMI=51.7 kg/m^2^ undergoing bariatric surgery; enoxaparin 30 mg BID | Lower incidence of VTE with 40 mg BID dosage (5.4% vs. 0.6%, p<.01) | Study was retrospective.  One of the few studies where the primary outcome was VTE rate. |
| Borkgren-Okonek, 2008 (60) | N= 223 patients undergoing bariatric surgery. Dosing was stratified based on BMI: ≤50 kg/m^2^= 40 mg BID (n=124; average BMI=44.9 kg/m^2^); > 50 kg/m2 = 60 kg/m^2^ (n=99; average BMI=57.4 kg/m^2^) | No placebo or control | 40 mg BID: Anti-Xa levels that were subtherapeutic (21%), therapeutic (79%) and supratherapeutic (0%)  60 mg BID: Anti-Xa levels that were subtherapeutic (14%), therapeutic (69%) and supratherapeutic (17%) | Study was open label.  There were a total of five major bleeding events (40 mg dose, n=4); 60 mg dose, n=1). Bleeding was not associated with a high anti-Xa level. |
| Rowan, 2008 (66) | N=33 patients with average BMI=48.5 kg/m^2^ undergoing bariatric surgery; enoxaparin 40 mg every 12 hours | N=19 patients with an average BMI=48.4 kg/m^2^ undergoing bariatric surgery; enoxaparin 30 mg every 12 hours | The percentage of patients with a therapeutic anti-Xa level in the 40 mg vs. 30 mg group was 31% vs. 0% (p=.01) after the first dose and 42% vs. 9% (p=.115) after the third dose | Study was prospective, non-randomized.  VTE rate not reported. A notable percentage of subtherapeutic anti-Xa levels was observed with both dosing regimens. |
| Simone, 2008 (68) | N=16 patients with an average BMI=47.3 kg/m^2^ who underwent bariatric surgery; enoxaparin 60 mg BID | N=24 patients with an average BMI=48.8 kg/m^2^ who underwent bariatric surgery; enoxaparin 40 mg BID | A greater percentage of patients remained subtherapeutic after the third dose in the 40 mg group (44% vs. 0%; p=.02). 57% of patients in the 60 mg group were supratherapeutic. | Study was prospective, non-randomized.  VTE rate not reported. There was one episode of significant bleeding which occurred in the 40 mg group. |
| Wang, 2014 (69) | N=1,559 hospitalized patients with a BMI ≥ 40 kg/m^2^ who received high-dose prophylaxis defined as 80 mg/day of enoxaparin or 22,500 units/day of unfractionated heparin | N=2,369 hospitalized patients with a BMI ≥ 40 kg/m^2^ who received standard prophylaxis (40 mg/day of enoxaparin or 10,000 – 15,000 units/day of unfractionated heparin. | VTE rate with high-dose was 0.77% vs. 1.48% [OR (95% CI) = 0.52 (0.27 – 1.00); p=.05]. No difference in bleeding (7.2% vs. 8.4%; p=.15) | Study was retrospective.  One of the few studies where the primary outcome was VTE rate. |
| Miranda, 2017 (63) | N=46 hospitalized patients with an average BMI=35.8 kg/m^2^; enoxaparin 60 mg daily | N=45 hospitalized patients with an average BMI=37.2 kg/m^2^; enoxaparin 40 mg daily | A greater percentage of patients had therapeutic anti-Xa levels in the 60 mg group (69% vs. 31%, p=.007). There were no VTE events in either group. | Study was prospective and randomized.  Population more so consistent with class II obesity vs. more extreme forms of obesity. |
| *Low-molecular weight heparin: Weight-based dosing strategies* | | | | |
| Rondina, 2010 (65) | N=28 medically ill patients with an average BMI = 48.1 kg/m^2^; enoxaparin 0.5 mg/kg/day using actual body weight | No placebo or control | Average peak anti-Xa level was 0.25 ± 0.11 IU/ml. No VTE or bleeding was detected. | Study was prospective.  There was no correlation with weight or BMI with peak anti-Xa level confirming the appropriateness of a weight-based approach. |
| Ludwig, 2011 (62) | N= 23 surgical ICU patients with an average  BMI= 46.4 kg/m^2^; enoxaparin 0.5 mg/kg twice daily using actual body weight | No placebo or control | 91% of patients had an appropriate anti-Xa level. There were no episodes of major bleeding. | Study was retrospective.  This study is specific to an ICU population. |
| Freeman, 2012 (61) | N=11 medically ill patients with an average BMI = 61.3 kg/m^2^; enoxaparin 0.5 mg/kg/day using actual body weight | N=11 medically ill patients with an average BMI = 63.4 kg/m^2^; enoxaparin 40 mg daily  N=9 medically ill patients with an average BMI =60.7 kg/m^2^; enoxaparin 0.4 mg/kg/day using actual body weight | Target anti-Xa levels achieved in significantly more patients receiving 0.5 mg/kg/day regimen compared the fixed dose or lower-weight-based regimen. | Study was prospective and randomized.  Average BMI was > 60 kg/m^2^ in all three dosing groups. Subtherapeutic anti-Xa levels were observed in 82%, 36% and 13% of the fixed dose, lower-weight based and higher-weight-based regimens, respectively. |
| Bickford, 2013 (59) | N=86 trauma patients with an average BMI = 35.3 kg/m^2^; enoxaparin 0.5 mg/kg twice daily using actual body weight. | No placebo or control | Target anti-Xa levels were achieved in 86% of patients. | Study was retrospective.  There were no bleeding complications.  There was no difference in anti-Xa levels between patients who did and did not develop a DVT (0.4 vs. 0.42 IU/ml). |
| Parikh, 2015 (64) | N=130 hospitalized patients with an average BMI = 45.6 kg/m^2^ (range 40 – 90.4 kg/m^2^); enoxaparin 0.5 mg/kg twice daily if very high risk or 0.5 mg/kg daily if moderate-high risk | No placebo or control | Target anti-Xa levels were achieved in 85% of levels drawn (120/141). | Study was retrospective.  There were 2 symptomatic VTE’s and 1 episode of major bleeding. |
| *Unfractionated heparin* | | | | |
| Samuel, 2015 (72) | N=141 neurocritical care patients with an average weight of 123 kg; heparin 7,500 units every 8 hours | N=257 neurocritical care patients with an average weight of 116 kg; heparin 5,000 units every 8 hours | No difference in VTE rate in the high dose (5.7%) vs. low dose (9.3%) groups, p=.2 | Study was retrospective.  On multivariate analysis, only time to initiation of heparin was significant for VTE. |
| Joy, 2016 (70) | N=751 hospitalized patients who weighed more than 100 kg; heparin 7,500 units every 8 hours | N=584 hospitalized patients who weighed more than 100 kg; heparin 5,000 units every 8 hours | No difference in VTE rates in the high dose (3%) vs. low dose (1.5%) groups, p=.14 | Study was retrospective.  VTE rate by BMI classification:  Obese class I: high dose (1%) vs. low dose (15%); p>.99  Obese class II: high dose (4%) vs. low dose (1%); p=.15  Obese class III: high dose (3%) vs. low dose (2%); p=.43 |
| Patanwala, 2018 (71) | N=1,673 hospitalized obese patients with an average BMI=37 kg/m^2^; heparin 5,000 units every 8 hours | N=3,437 hospitalized non-obese patients with an average BMI=24 kg/m^2^; heparin 5,000 units every 8 hours | No difference in VTE rates between obese and non-obese patients (0.7% vs. 0.6%, p=.7) | Study was retrospective.  In the subgroup of patients in the ICU, there was no difference in VTE rate [obese (n=409), 2.2% vs. non-obese (n=897), 1.7%; p=.51] |
| Mason, 2019 (73) | N=190 hospitalized obese patients with an average BMI=49 kg/m^2^; heparin 7,500 units every 8 hours | N=115 hospitalized obese patients with an average BMI=51 kg/m^2^; enoxaparin 40 mg every 12 hours | No difference in VTE rates between heparin and enoxaparin (2.1% vs. 0.9%, p=.388)  Major bleeding rates were higher with heparin (33% vs. 21%, p=.025) | Study was retrospective.  Patients in the heparin group were older, had higher baseline creatinine values, concomitant antiplatelet use and more were surgical patients. |

BMI = body mass index; VTE = venous thromboembolism; DVT = deep vein thrombosis; BID = twice daily, ICU = intensive care unit
